# Wdr1-Dependent Actin Reorganization in Platelet Activation

**DOI:** 10.1371/journal.pone.0162897

**Published:** 2016-09-14

**Authors:** Swapan K. Dasgupta, Anhquyen Le, Qi Da, Miguel Cruz, Rolando E. Rumbaut, Perumal Thiagarajan

**Affiliations:** 1 Department of Pathology and Immunology, Baylor College of Medicine, Houston, Texas, United States of America; 2 Department of Medicine, Baylor College of Medicine, Houston, Texas, United States of America; 3 Center for Translational Research on Inflammatory Diseases (CTRID), Michael E. DeBakey Veterans Affairs Medical Center, Houston, Texas, United States of America; Katholieke Universiteit Leuven, BELGIUM

## Abstract

In resting platelets, the integrin αIIbβ3 is present in a low-affinity “bent” state. During platelet aggregation, intracytoplasmic signals induce conformational changes (inside-out signaling) that result in a “swung-out” conformation competent to bind ligands such as fibrinogen. The cytoskeleton plays an essential role in αIIbβ3 activation. We investigated the role of the actin interacting protein Wdr1 in αIIbβ3 activation. Wdr1-hypomorphic mice had a prolonged bleeding time (> 10 minutes) compared to that of wild-type mice (2.1 ± 0.7 minutes). Their platelets had impaired aggregation to collagen and thrombin. In a FeCl_3_ induced carotid artery thrombosis model, vessel occlusion in Wdr1-hypomorphic mice was prolonged significantly compared to wild-type mice (9.0 ± 10.5 minutes versus 5.8 ± 12.6 minutes (p = 0.041). Activation-induced binding of JON/A (a conformation-specific antibody to activated αIIbβ3) was significantly less in Wdr1-hypomorphic platelets at various concentrations of collagen, indicating impaired inside-out activation of αIIbβ3, despite a normal calcium response. Actin turnover, assessed by measuring F-actin and G-actin ratios during collagen- and thrombin-induced platelet aggregation, was highly impaired in Wdr1-hypomorphic platelets. Furthermore, talin failed to redistribute and translocate to the cytoskeleton following activation in Wdr1-hypomorphic platelets. These studies show that Wdr1 is essential for talin-induced activation of αIIbβ3 during platelet activation.

## Introduction

Integrin αIIbβ3 (glycoprotein IIb-IIIa complex) is a noncovalent heterodimeric transmembrane cell adhesion molecule in platelets. It mediates aggregation by binding to bivalent ligands such as fibrinogen and von Willebrand factor on the platelet membrane [1, 2]. In resting platelets, αIIbβ3 is present in a low-affinity “bent” conformation on the cell surface. Platelet activation results in conformational changes (“inside-out signaling”) in αIIbβ3, inducing the receptor to adopt a “swung-out” conformation with an increased binding affinity for ligands. The linkage of the actin cytoskeleton to αIIbβ3 during platelet activation plays an essential role in the conformational changes in αIIbβ3. The cytosolic protein talin mediates this interaction between αIIbβ3 and cytoskeleton [3, 4]. In resting platelets, talin is distributed throughout the cytoplasm. Upon activation, a significant amount of talin rapidly redistributes to a peripheral, submembranous location [4, 5] and interacts with β integrin [3] The regulation of talin’s interaction with αIIbβ3 is actively pursued, as it is the final common step in αIIbβ3 activation. A talin mutant (L325R) has been described that binds to actin cytoskeleton but fails to activate αIIbβ3 showing that binding to actin cytoskeleton precedes αIIbβ3 activation [6].

Talin, a 270-kDa large dimeric cytosolic protein, consists of a flexible rod domain and a globular head containing a FERM (protein 4.1, ezrin, radixin, moesin) domain comprising 4 subdomains F0, F1, F2, and F3. The rod domain consists of amphipathic α-helices that are assembled into 5-helix bundles [7, 8]. The talin head domain binds to β3 cytoplasmic domain to induce the “swung-out” conformation [3]. In resting platelets, binding to αIIbβ3 is constrained by the interaction of the head and rod domains [9]. This auto-inhibitory interaction between the head and the rod domains regulates the function of talin and it is disrupted by activation-induced conformational changes in talin. Several proteins have been identified as the cue for talin activation such as the hematopoiesis-restricted adapter protein, ADAP [10], the Rap1-GTP interacting adapter molecule [11], G protein Gα13 [12] and possibly many others.

During platelet activation, there is a marked morphological change due to rapid reorganization of the cortical actin cytoskeleton due to cofilin1-induced actin turnover. Wdr1, the mammalian homolog of Aip1 (actin interacting protein 1 in yeast), enhances cofilin's capacity to accelerate depolymerization of actin [13–15]. Hemostasis is defective in Wdr1-hypomorphic mice. We present evidence that Wdr1-mediated actin reorganization is one of the essential steps for the talin-induced conformational changes in β3 integrins during platelet activation.

## Materials and Methods

### Reagents

Collagen (equine tendon collagen) was purchased from Helena Laboratories and anti-β-actin antibody was purchased from Cell Signaling Technology. Anti-Wdr1, anti-GAPDH, anti-β3 integrin, anti-β-actin and anti-talin antibodies were obtained from Santa Cruz Biotechnology, while Alexa Fluor-labeled secondary antibodies were purchased from Life Technologies. Antibodies for flow cytometry were purchased from BD biosciences (β3, αIIb and β1 integrins), E bioscience (α2 integrin)), R&D systems (platelet glycoprotein VI, GP VI) and Emfret analytics (JON/A). Reagents for proximity ligation assays (Duolink) were from Sigma Aldrich. All other chemicals were purchased from Sigma Aldrich. The hypomorphic allele of Wdr1 mice has been described before [16]. The mutant mouse has a T>A transversion in the second dinucleotide of the intron 9 splice donor and it produces a mutant transcript containing a 6-bp in-frame deletion that results in a incorrectly folded, nonfunctional protein [16]. The small amount of normal splicing that occurs produces some wild-type protein, resulting in a hypomorphic allele. The Institutional Animal Care and Use Committee of Baylor College of Medicine approved all animal protocols including the euthanasia procedure with inhalational isoflurane surgical plane anesthesia followed by cervical dislocation. The Wdr1 hypomorphic mice were developed at Baylor College of Medicine [16] and C57BL6 were from Jackson Laboratories.

### Isolation of Platelets and endothelial cells

Blood was obtained from the inferior vena cava into ACD (acid-citrate-dextrose) anticoagulant from ≥4-month-old mice under isoflurane anesthesia. Blood was diluted with an equal volume of modified Tyrode’s buffer (137 mM NaCl, 2.7 mM KCl, 5 mM Hepes, 1 mM MgCl_2_, 3 mM NaH_2_PO_4_, and 5.5 mM Dextrose, pH, 7.4) containing 0.5% bovine serum albumin (BSA). Platelet-rich plasma was obtained by centrifugation at 75 g for 15 minutes. Prostaglandin E1 (1 μM) and apyrase (0.5 unit) were added, and platelets were sedimented by centrifugation at 600 g for 10 minutes, washed once in Tyrode’s buffer containing apyrase and suspended in Tyrode’s buffer. Platelets were counted in a Coulter counter and adjusted to 2 x10^8^ platelets/ml for aggregation studies [17]. The mean platelet volume was determined in a hematology analyzer (ABX Micros 60). Pulmonary microvascular endothelial cells were isolated as described previously [18].

### Calcium Studies

Briefly, washed platelets (10^8^/ml) from Wdr1-hypomorphic mice or littermate controls were incubated with Fura-2AM (12.5 μM) for 30 minutes at 37°C, washed with modified Tyrode’s buffer twice and finally resuspended in the same buffer containing 1.5 mM calcium chloride. Ca^2+^ measurements were performed in a 96 well plate containing 200 μL of platelet suspension using a SynergyMX (BioTek Instruments) spectrophotometer with an excitation wavelength alternating from 340 to 380 nm while the emission wavelength was set at 510 nm. When a stable baseline was achieved, collagen (10 μg/ml) or thrombin (0.1 unit/ml) was added and changes in fluorescence were recorded. Concentration of intracellular free calcium was determined by the formula of Grynkiewicz et al. [19].

### Ferric chloride–induced thrombosis

Ferric chloride (FeCl_3_)-induced thrombosis was performed as described previously [20] by applying a 1.2 × 1.2-mm piece of filter paper soaked in 7.5% FeCl_3_ to the common carotid artery of an anesthetized mouse. After 3 minutes of application, the filter paper was removed and the artery rinsed with isotonic saline. The time to vessel occlusion was determined using a Doppler flow probe. The tail bleeding time was performed as described before [17].

### Immunofluorescence studies

Platelets were immobilized on a polylysine-coated cover slip (resting platelets) or on a collagen-coated cover slip (activated platelets) for at 37°C for 15 minutes. The platelets were fixed, permeabilized with 0.1%triton X-100, blocked with 5% BSA and incubated with 1:200 dilution of anti talin antibody and anti β3 integrin overnight at 4°C or Alexa 488-phalloidin for one hour at room temperature. For talin and β3 integrin, the cover slips were washed and incubated with appropriate secondary antibody conjugated to Alexa 647 (talin) or Alexa 488 (β3 integrin). Images were taken in a DeltaVision OMX microscope (GE Healthcare) at 1000× magnification. The images were acquired with an Olympus 100x/1.40na UPLSAPO oil immersion objective with z step of 0.15um. The camera is a CoolSNAP HQ2 (Photometrics). Images were deconvolved using SoftWoRX 6.5.2 that applies a 3D iterative constrained deconvolution algorithm (ratio). The iteration number was 10.

### Platelet activation, lysis and fractionation of platelets

Platelets were incubated with collagen (10 μg/ ml) at 37° C in presence of calcium (1.5 mM) in an aggregometer. At various time, platelets were lysed with an equal volume of 2 × lysis buffer containing 100 mM Tris-HCl (pH 7.4), 2% Triton X 100 containing protease and phosphatase inhibitor cocktail (Thermo scientific) and kept on ice for 15 minutes. The lysate was centrifuged at 15600 g to obtain cytoskeleton, and soluble cytosolic fractions [21]

### F-actin/G-actin ratio and F actin content

The ratio of filamentous actin (F-actin) to monomeric actin (G-actin) was determined by immunoblots. Washed platelets (0.5 x 10^8^/ml) were activated with collagen (10 μg/ml) ot thrombin (0.05 unit/ml) and lysed with an equal volume of 2 × lysis buffer containing 100 mM Tris-HCl (pH 7.4), 10 mM EGTA, 2 mM MgCl_2_ and 2% Triton X 100 containing protease and phosphatase inhibitor cocktail (Thermo scientific) and kept on ice for 15 minutes. Lysates were centrifuged at 100,000 g for 1 hour to separate Triton-X-100-soluble G-actin from Triton-X-100-insoluble F-actin. Supernatant containing G-actin was collected, and the F-actin pellets were washed twice in cold lysis buffer and solubilized in 8 M urea. The supernatants and the pellets were reconstituted to the same initial volume. Fractions were proportionally loaded onto SDS-polyacrylamide gels, electrophoresed and transferred onto PVDF membrane for probing with an anti-actin antibody. Densitometric quantification of the western blot was used to determine the F-actin versus G-actin content. The F-actin to G-actin ratio of resting platelets was considered as 1. FITC-phalloidin binding to platelets was also used to quantify F-actin content as previously described [22]. Resting and activated platelets were fixed with 4% paraformaldehyde, permeabilized with 0.5% Triton X-100, and stained with 10 μM FITC-phalloidin for 1 hour. FITC fluorescence was monitored by flow cytometry.

### Proximity Ligation Assay

Spatial colocalization of talin and β3 integrin were analyzed with proximity ligation assay (PLA) technology in washed platelets using Duolink in situ PLA detection kit orange (Sigma), following manufacturer’s instructions. Platelets from Wdr-1-deficient and wild-type were isolated, activated with collagen (10 μg/ml) and immobilized on polylysine coated platelets. Platelets were then fixed, permeabilized and blocked for 30 minutes at room temperature with 0.5% BSA in PBS and incubated with primary antibodies (mouse anti-β3 and rabbit anti-talin (the same antibodies used for immunofluorescence assays) overnight at 4°C. Cells were labelled with Duolink PLA anti-rabbit PLUS and anti-mouse MINUS probes as per manufacture’s instruction. The orange fluorescent fluorophore tagged oligonucleotides were visualized using a confocal microscope.

### Statistical Analysis

All data are expressed as mean ± standard deviation of triplicate experiments except when indicated otherwise. Comparisons between individual groups were performed using the Student T-test with paired and unpaired samples. Data not distributed normally were analyzed with a Mann-Whitney U test. A probability value (P) of 0.05 or below was considered statistically significant.

## Results

### Wdr1-hypomorphic mice

**Fig 1:**
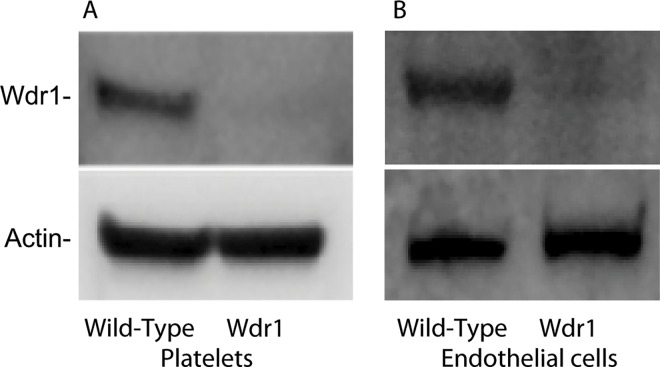
Wdr1 expression. Platelets (Panel A) and pulmonary endothelial cells (Panel B) from wild-type and Wdr1-hypomorphic mice were isolated, solubilized in 1% SDS and subjected to PAGE followed by immunoblot with antibodies to Wdr1 or β actin.

The phenotype of mice homozygous for a hypomorphic allele of Wdr1 (Wdr1^rd/rd^) mouse has been described [16]. Wdr1-hypomorphic mice exhibit spontaneous autoinflammatory disease and thrombocytopenia. These mice do not exhibit spontaneous bleeding. The amount of Wdr1 protein in Wdr1^rd/rd^ mice is decreased both in platelets (Fig 1, panel A) and in pulmonary microvascular endothelial cells (Fig 1, panel B). The platelet counts in Wdr1-hypomorphic mice are one-third of their wild-type controls (0.4 ± 0.2 × 10^6^/μl) for Wdr1-hypomorphic mice versus 1.2 ± 0.5 × 10^6^ μl for wild-type controls and the mean platelet volume is increased (7.0 ± 0.9 fl for Wdr1 deficient mice versus 5.33 ± 0.4 fl for wild-type, N = 10 and P = 0.0002).

### Defective platelet aggregation in Wdr1-hypomorphic mice

**Fig 2:**
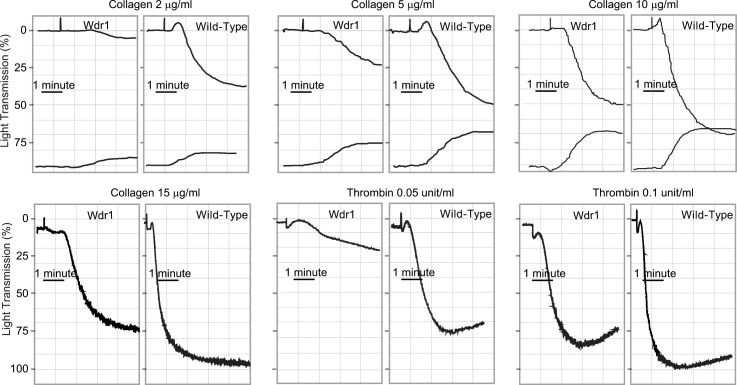
Platelet aggregation in Wdr1-hypomorphic mice. Platelets were isolated from both Wdr1-hypomorphic mice and littermate wild-type controls and stimulated with various concentrations of collagen (Panels A-D) or thrombin (Panels E and F). Aggregations were measured using a turbidometric aggregometer. In the upper panel, the top tracings reflect platelet aggregation while the bottom tracings indicate ATP secretion. The traces shown are representative of at least 3 or more independent experiments.

The aggregation response of Wdr1-hypomorphic mice to collagen showed impairment compared to the wild-type controls at various concentrations of collagen (Fig 2, Panels A-D). There was virtually no platelet shape change with collagen in Wdr1-hypomorphic mice compared to wild-type controls. The secretion of ATP in response to collagen was similar to wild-type controls (Fig 2, Panels A-C). The aggregation response to thrombin was also impaired in Wdr1-hypomorphic mice (Fig 2, Panels E and F). Even at higher concentrations of thrombin, aggregation showed impairment compared to the controls.

**Fig 3:**
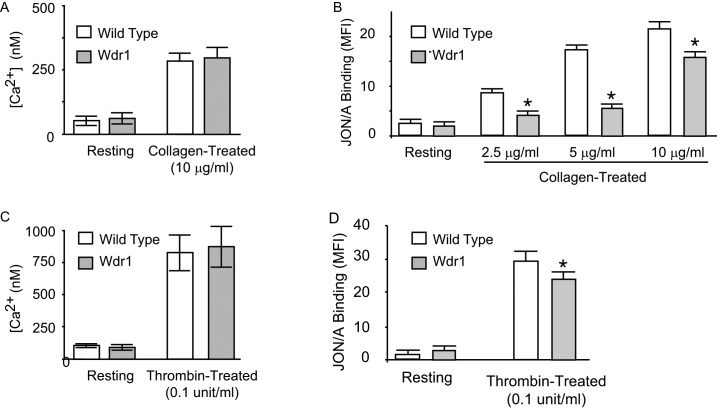
Collagen-induced calcium signaling and conformational changes in αIIbβ3. *Panel A and C*. Intracellular Ca^2+^ levels in resting and activated platelets (n = 3/group). Platelets from Wdr1-hypomorphic and littermate wild-type mice were loaded with Fura-2, washed and suspended in Tyrode’s buffer for 5 minutes before the addition of collagen or thrombin. The maximal increase in calcium levels were quantified by Fura-2 fluorescence. Shown are the mean and standard deviations of three separate experiments are shown. *Panel B and D*. Isolated platelets were stimulated with various concentrations of collagen (0–10 μg/mL) and thrombin (0.1unit/ml). Conformational changes in αIIbβ3 were assessed by flow cytmetry using activation-specific antibody, JON/A-PE. The mean fluorescence index (MFI) and standard deviation of three separate experiments are shown. * denotes P <0.05

Since impaired surface expression of innate receptors could result in the observed hemostatic defects, we determined the expression of platelet glycoprotein Ibα, integrins subunits αIIb, β1 and β3, and glycoprotein IV in Wdr1-hypomorphic mice and their wild-type controlsby flow cytometry (mean fluorescence intensity). Similar levels of surface expression were observed in Wdr1-hypomorphic platelets as in wild-type control (Table 1). The rise in intraplatelet Ca^2+^, following agonist binding, has been shown to play an essential role in the activation and in the recruitment of feedback signaling mechanisms such as ADP secretion. We measured the calcium response to collagen stimulation. As shown in Fig 3, panel A, collagen stimulation increased Ca^2+^ levels in both wild-type and Wdr1-hypomorphic platelets to a similar extent. These data indicate that the defective platelet aggregation in Wdr1-hypomorphic mice is downstream of increased intraplatelet Ca^2+^.

**Table 1:** Expression of platelet glycoproteins in wild-type littermate control and Wdr1-hypomorphic mice. Washed platelets were incubated with fluorescein-labeled antibodies to glycoproteins and the mean fluorescence (MFI) intensities are measured.

Glycoprotein	Wild-type	Wdr1-hypomorphic
β3 integrin	38 ± 4.3	35.1± 3.5
αIIb integrin	51 ± 2.2	53.7 ± 2.3
α2 integrin	21.1 ± 1.1	23.4 ± 2.3
β1 integrin	31.2 ± 1.5	32.3 ±0.8
GPVI	34.2 ± 0.9	31.6 ± 1.8

### Defective αIIbβ3 activation in Wdr1-hypomorphic platelets

To assess agonist-induced activation of integrin αIIbβ3 in Wdr1-hypomorphic platelets, we measured the conformational change in αIIbβ3 by flow cytometry using activation-specific αIIbβ3 antibody, JON/A-PE, in response to various concentrations of collagen. Wdr1-hypomorphic mice had an impairment in binding indicating that the activation of αIIbβ3, which is the penultimate step in platelet aggregation, is defective (Fig 3, Panel B).

### In vivo hemostasis in Wdr1-hypomorphic mice

**Fig 4:**
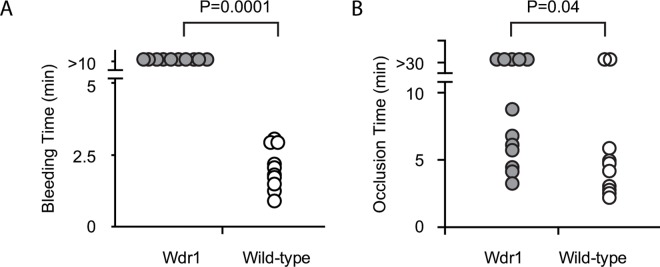
Effect of Wdr1-deficiency on hemostasis in vivo. Panel A. Tail bleeding time. The data represent 10 mice in each group. Panel B. Time for total occlusion. Flow cessation was determined by intravital microscopy following endothelial injury induced by ferric chloride in carotid arteries. The data represents Wdr1-hypomorphic (n = 12) and wild-type mice (n = 10). The p values were determined by nonparametric Mann-Whitney U test).

We assess the *in vivo* significance of these observations by measuring tail-bleeding time. The tail bleeding time of Wdr1-hypomorphic mice was prolonged significantly, (> 10 minutes) compared to wild-type mice (2.1 ± 0.7 min) (Fig 4, Panel A). Furthermore, in a FeCl_3_ induced carotid artery injury/thrombosis model, vessel occlusion time in Wdr1-hypomorphic was also significantly prolonged compared to wild-type mice (15.8 ± 12.6 minutes versus 9.0 ± 10.5 minutes (p = 0.041) (Fig 4, Panel B). These data underscore the role of Wdr1 in hemostasis in vivo. Hemostatic defects observed in Wdr1-hypomorphic mice are not ascribable to differences in platelet counts because occlusive thrombus formation in chemically injured carotid artery is only impaired when platelet counts drop to <10% of control [23].

### Activation-induced redistribution of talin

**Fig 5:**
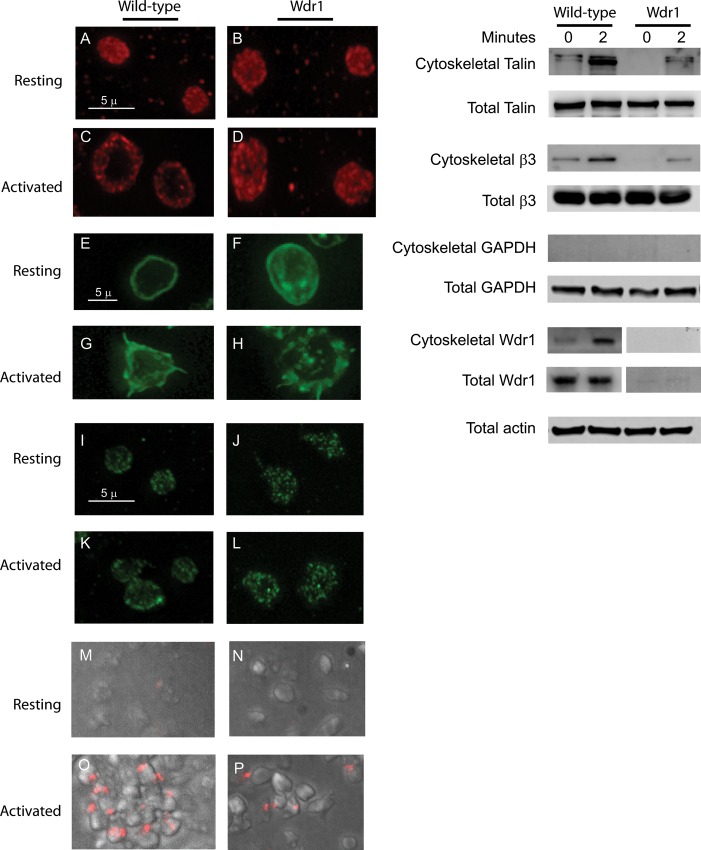
Activation-induced redistribution of talin in platelets. Left Panels. Washed platelets from wild-type (Panels A, C, E, G, I and K) or Wdr1-hypomorphic mice (Panels B, D, F, H, J and L) were immobilized on polylysine (resting) or collagen (activated) coated coverslips as described in the experimental section. The platelets were incubated with talin-1 antibody (Panels A-D) or phalloidin (Panels E-H), and β3 antibody (Panels I-L) examined under a fluorescence microscope with 1000x magnification. Panels M-P. β3 integrin and talin Interaction was assessed by proximity ligation assay in resting and collagen treated platelet from wild-type and Wdr1-hypomorphic mice. Right Panels. Washed platelets from wild-type or Wdr1-hypomorphic mice were treated with collagen (10 μg/ml) in suspension. The cytoskeletal fractions from resting (time 0 minute) and collagen-treated platelets (time 2 minutes) in suspension were isolated by centrifugation at 15600g, washed, solubilized, subjected to SDS-PAGE and immunoblotted with antibodies to talin, integrin β3, GAPDH, Wdr1 or actin. The purity of cytoskeleton fractions were assessed by the presence of GAPDH. actin, integrin β3 and talin of the total cell lysate are shown as loading control.

In resting unstimulated wild type platelets, talin is localized primarily to the cytoplasm. However following activation, it was recruited to a peripheral, submembranous location, where it interacts with the cytoplasmic domains of αIIbβ3 (Fig 5, Left Panels A and C). In Wdr1-hypomorphic platelets, the translocation of talin, from the cytosol to the cell membrane is impaired (Fig 5, Left Panels B and D). F-actin is increased in resting and activated platelets. F-actin was predominantly observed in the cortical region of wild type platelets whereas in Wdr1-hypomorphic platelets, in addition to the cortical actin, more F-actin is seen in the cytosol also (Fig 5 Panels E-H). There was no significant difference in the distribution of αIIbβ3 following activation in both resting and activated platelets between Wdr1-hypomorphic and the wild-type mice (Fig 5, Left Panels I-L). The association of β3 integrin with talin following activation was assessed by the proximity ligation assay. In Wdr1-hypomorphic platelets, the association of β3 integrin with talin was impaired (Fig 5, Left Panels M-P). The puncta represents the association in close proximity (<40 nm). In Wdr1-hypomorphic platelets, the translocation of talin to actin cytoskeleton was impaired (Fig 5, Right Panel). Following translocation, talin anchors αIIbβ3 to the cytoskeleton and as expected there was defective association of αIIbβ3 to the cytoskeleton in Wdr1-hypomorphic mice (Fig 5, Right Panel). Interestingly Wdr1, which was normally localized to the cell membrane, associated with platelet cytoskeleton following platelet activation (Fig 5, Right Panel).

### Wdr1-hypomorphic mice have defective actin turnover

**Fig 6:**
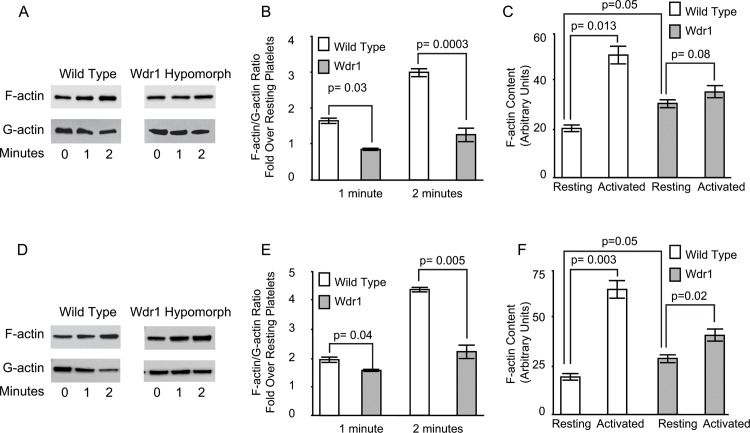
Actin Reorganization in platelets. Panels A and D. Washed platelets in suspension were stimulated with 10 μg/ml of collagen (Panel A) or 0.05 unit/ml of thrombin (Panel D), lysed and centrifuged. The supernatant containing the soluble G-actin and triton X-100-insoluble pellet containing F-actin were solubilized, and subjected to SDS-PAGE and immunoblotted using monoclonal anti-actin antibody. *Panels B and E*. The immunoblots of actin were quantified and a change in the ratio of insoluble F-actin to triton X-100-soluble G-actin is a considered an indicator of actin reorganization. The mean and standard deviations of three independent experiments are shown. *Panels C and F*. Washed platelets in suspension were stimulated with 10 μg/ml of collagen (Panel C) or 0.05 unit/ml of thrombin (Panel F), for 2 minutes, fixed with 4% paraformaldehyde, permeabilized with 0.5% Triton X-100, and stained with Alexa488-phalloidin. The fluorescence was measured by flow cytometry and expressed as fluorescence intensity in resting and collagen treated platelets. The mean and standard deviations of three independent experiments are shown.

We assessed the activation-induced actin turnover following platelet activation with 10 μg/ml of collagen or 0.05 units/ml of thrombin by quantifying the ratio of F-actin to G-actin. As shown in Fig 6, Panels A-B and D-E, there was a significant impairment in actin turnover in Wdr1-hypomorphic platelets compared to wild-type platelets as determined by F-actin/G-actin ratio, consistent with the fact that Wdr1-enhanced cofilin1-induced actin turnover. Although there was increased F-actin in resting Wdr1-hypomorphic platelets there was virtually no actin turnover in the first minute following collagen or thrombin stimulation and there was only a modest increase at two minutes (Fig 6, Panels B and E). We also saw similar results when we quantified F-actin by flow cytometry with FITC-phalloidin in collagen and thrombin stimulated platelets (Fig 6, Panels C and F). These findings show that de novo actin reorganization, which occurs during platelet activation, is impaired in Wdr1-hypomorphic mice.

## Discussion

The linkage of the actin cytoskeleton to αIIbβ3 plays an essential role in platelet aggregation and in adhesion to subendothelial matrix components. The actin cytoskeleton is in a dynamic equilibrium between two states–globular monomeric G-actin and filamentous polymeric F-actin. In resting platelets, actin is present as a submembranous latticework of short cross-linked actin filaments, known as the membrane skeleton [24]. Platelet activation results in depolymerization and reorganization into long dense F-actin fibers, which course through the body of the platelet and it can be sediment at 15,600 g. Turnover of actin is highly regulated and a large number of actin binding proteins are present to mediate the assembly, disassembly, and rearrangement of the cytoskeleton. ADF/cofilin family of actin-binding proteins disassembles actin filaments. Platelets contain cofilin1 [25], which changes the twist of F-actin filament and severs filamentous F-actin without capping it [26]. This severing facilitates new actin fiber formation via the newly exposed barbed ends. Wdr1 is the mammalian equivalent of actin interacting protein-I in yeast, where it binds to the cofilin–F-actin complex and strongly enhances the severing activity of cofilin [15] and thus contributes to the maintenance of high concentrations of actin monomers for actin reorganization [27]. Recent evidences show that Wdr-1-dependent actin turnover in the maintenance cell adhesion in intestinal epithelial apical junctions [28] and in the cell shape and planar orientation of epidermal cells [29]. Our data show in platelets that Wdr-1 also plays a crucial role in integrin activation in platelets essential for aggregation and spreading. Despite the defects, Wdr1-hypomorphic mice do not have spontaneous bleeding. Actin polymerization is a versatile process and involves in several cellular events such as migration, endocytosis and cell division and multiple redundant pathways are operative [30]. Because of the large number of actin-binding proteins that influence these processes, deficiency of one factor will be readily compensated.

In Wdr1-hypomorphic platelets, activation-induced talin redistribution and association of talin with the cytoskeleton is impaired. Talin, a mechanosensitive protein, can sense forces generated by physical stretching [9, 31]. Previous studies have shown that application of force up to 20 pN with an atomic force microscope tip can stretch the rod domain of talin by 100 nm. This stretching exposes cryptic binding sites for vinculin that reinforces talin association with cytoskeleton [32, 33]. The head domain of talin interacts with integrin αIIbβ3 [34] and two distinct sites on the cytoplasmic domain of αIIbβ3 were identified as binding sites, a membrane distal phosphotyrosine domain and a second unique membrane proximal region [35]. Talin binding to αIIbβ3 is sufficient to induce conformational changes necessary for fibrinogen binding. In resting platelets, talin binding to αIIbβ3 is constrained by the interaction of the head with the rod domain [9]. Platelet activation releases the constraints allowing talin to bind αIIbβ3. Previous work has shown that actin filament polymerization is necessary for fibrinogen binding to αIIbβ3 [36, 37]. The mechanism by which actin turnover regulates talin function is not clear. Talin contains several actin-binding domains: one in the FERM domain and at least two in the rod domain, including a conserved five-helix bundle actin-binding domain [38]. Talin binds to both G- and F-actin [39]. Talin is bound to G-actin in the cytosol in resting platelets. We speculate that forces generated by polymerization of actin during platelet activation can be transmitted to talin, which can unfold in force-dependent manner to expose additional binding sites for F-actin and αIIbβ3. A recent study [40], using reconstituted actomyosin complex, demonstrated that the complex induces a positive feedback to reinforce the actin–talin–vinculin association. Taken together, these studies show that Wdr1-mediated actin turnover and cytoskeletal reorganization play a role in modulating talin interaction with cytoskeleton and subsequently to αIIbβ3.

## References

[1] Coller BS. alphaIIbbeta3: structure and function. J Thromb Haemost. 2015;13 Suppl 1:S17–25. Epub 2015/07/08. 10.1111/jth.12915 . 26149019 PMC4888797

[2] Ginsberg MH. Integrin activation. BMB reports. 2014;47(12):655–9. Epub 2014/11/13. 25388208 10.5483/BMBRep.2014.47.12.241 PMC4345508

[3] Goksoy E, Ma YQ, Wang X, Kong X, Perera D, Plow EF, et al Structural basis for the autoinhibition of talin in regulating integrin activation. Molecular cell. 2008;31(1):124–33. Epub 2008/07/11. 10.1016/j.molcel.2008.06.011 18614051 PMC2522368

[4] Song X, Yang J, Hirbawi J, Ye S, Perera HD, Goksoy E, et al A novel membrane-dependent on/off switch mechanism of talin FERM domain at sites of cell adhesion. Cell research. 2012;22(11):1533–45. Epub 2012/06/20. 10.1038/cr.2012.97 22710802 PMC3494399

[5] Bertagnolli ME, Locke SJ, Hensler ME, Bray PF, Beckerle MC. Talin distribution and phosphorylation in thrombin-activated platelets. Journal of cell science. 1993;106 (Pt 4):1189–99. Epub 1993/12/01. . 8126100 10.1242/jcs.106.4.1189

[6] Haling JR, Monkley SJ, Critchley DR, Petrich BG. Talin-dependent integrin activation is required for fibrin clot retraction by platelets. Blood. 2011;117(5):1719–22. Epub 2010/10/26. 10.1182/blood-2010-09-305433 20971947 PMC3056596

[7] Critchley DR. Biochemical and structural properties of the integrin-associated cytoskeletal protein talin. Annual review of biophysics. 2009;38:235–54. Epub 2009/05/07. 10.1146/annurev.biophys.050708.133744 . 19416068

[8] Choquet D, Felsenfeld DP, Sheetz MP. Extracellular matrix rigidity causes strengthening of integrin-cytoskeleton linkages. Cell. 1997;88(1):39–48. Epub 1997/01/10. . 9019403 10.1016/s0092-8674(00)81856-5

[9] Goult BT, Xu XP, Gingras AR, Swift M, Patel B, Bate N, et al Structural studies on full-length talin1 reveal a compact auto-inhibited dimer: implications for talin activation. Journal of structural biology. 2013;184(1):21–32. Epub 2013/06/04. 10.1016/j.jsb.2013.05.014 23726984 PMC3799832

[10] Kasirer-Friede A, Kang J, Kahner B, Ye F, Ginsberg MH, Shattil SJ. ADAP interactions with talin and kindlin promote platelet integrin alphaIIbbeta3 activation and stable fibrinogen binding. Blood. 2014;123(20):3156–65. Epub 2014/02/14. 10.1182/blood-2013-08-520627 24523237 PMC4023421

[11] Lee HS, Lim CJ, Puzon-McLaughlin W, Shattil SJ, Ginsberg MH. RIAM activates integrins by linking talin to ras GTPase membrane-targeting sequences. J Biol Chem. 2009;284(8):5119–27. Epub 2008/12/23. 10.1074/jbc.M807117200 19098287 PMC2643525

[12] Srinivasan S, Schiemer J, Zhang X, Chishti AH, Le Breton GC. Galpha13 Switch Region 2 Binds to the Talin Head Domain and Activates alphaIIbbeta3 Integrin in Human Platelets. J Biol Chem. 2015;290(41):25129–39. Epub 2015/08/21. 10.1074/jbc.M115.650978 26292217 PMC4599016

[13] Okada K, Obinata T, Abe H. XAIP1: a Xenopus homologue of yeast actin interacting protein 1 (AIP1), which induces disassembly of actin filaments cooperatively with ADF/cofilin family proteins. Journal of cell science. 1999;112 (Pt 10):1553–65. Epub 1999/04/23. . 10212149 10.1242/jcs.112.10.1553

[14] Rodal AA, Tetreault JW, Lappalainen P, Drubin DG, Amberg DC. Aip1p interacts with cofilin to disassemble actin filaments. J Cell Biol. 1999;145(6):1251–64. Epub 1999/06/15. 10366597 10.1083/jcb.145.6.1251 PMC2133144

[15] Ono S. Regulation of actin filament dynamics by actin depolymerizing factor/cofilin and actin-interacting protein 1: new blades for twisted filaments. Biochemistry. 2003;42(46):13363–70. Epub 2003/11/19. 10.1021/bi034600x . 14621980

[16] Kile BT, Panopoulos AD, Stirzaker RA, Hacking DF, Tahtamouni LH, Willson TA, et al Mutations in the cofilin partner Aip1/Wdr1 cause autoinflammatory disease and macrothrombocytopenia. Blood. 2007;110(7):2371–80. Epub 2007/05/23. 10.1182/blood-2006-10-055087 17515402 PMC1988957

[17] Dasgupta SK, Le A, Haudek SB, Entman ML, Rumbaut RE, Thiagarajan P. Rho associated coiled-coil kinase-1 regulates collagen-induced phosphatidylserine exposure in platelets. PLoS One. 2013;8(12):e84649 Epub 2013/12/21. 10.1371/journal.pone.0084649 24358370 PMC3865301

[18] Sobczak M, Dargatz J, Chrzanowska-Wodnicka M. Isolation and culture of pulmonary endothelial cells from neonatal mice. Journal of visualized experiments: JoVE. 2010;(46). Epub 2010/12/24. 10.3791/2316 21178973 PMC3278331

[19] Grynkiewicz G, Poenie M, Tsien RY. A new generation of Ca2+ indicators with greatly improved fluorescence properties. J Biol Chem. 1985;260(6):3440–50. . 3838314

[20] Dasgupta SK, Le A, Chavakis T, Rumbaut RE, Thiagarajan P. Developmental endothelial locus-1 (Del-1) mediates clearance of platelet microparticles by the endothelium. Circulation. 2012;125(13):1664–72. Epub 2012/03/06. 10.1161/CIRCULATIONAHA.111.068833 . 22388320

[21] Fox JE, Lipfert L, Clark EA, Reynolds CC, Austin CD, Brugge JS. On the role of the platelet membrane skeleton in mediating signal transduction. Association of GP IIb-IIIa, pp60c-src, pp62c-yes, and the p21ras GTPase-activating protein with the membrane skeleton. J Biol Chem. 1993;268(34):25973–84. Epub 1993/12/05. . 7503992

[22] Hartwig JH, Kung S, Kovacsovics T, Janmey PA, Cantley LC, Stossel TP, et al D3 phosphoinositides and outside-in integrin signaling by glycoprotein IIb-IIIa mediate platelet actin assembly and filopodial extension induced by phorbol 12-myristate 13-acetate. J Biol Chem. 1996;271(51):32986–93. Epub 1996/12/20. . 8955143 10.1074/jbc.271.51.32986

[23] Morowski M, Vogtle T, Kraft P, Kleinschnitz C, Stoll G, Nieswandt B. Only severe thrombocytopenia results in bleeding and defective thrombus formation in mice. Blood. 2013;121(24):4938–47. Epub 2013/04/16. 10.1182/blood-2012-10-461459 . 23584880

[24] Fox JE. Cytoskeletal proteins and platelet signaling. Thromb Haemost. 2001;86(1):198–213. Epub 2001/08/07. . 11487008

[25] O'Neill EE, Brock CJ, von Kriegsheim AF, Pearce AC, Dwek RA, Watson SP, et al Towards complete analysis of the platelet proteome. Proteomics. 2002;2(3):288–305. Epub 2002/03/29. . 11921445 10.1002/1615-9861(200203)2:3<288::aid-prot288>3.0.co;2-0

[26] McGough A, Pope B, Chiu W, Weeds A. Cofilin changes the twist of F-actin: implications for actin filament dynamics and cellular function. J Cell Biol. 1997;138(4):771–81. Epub 1997/08/25. 9265645 10.1083/jcb.138.4.771 PMC2138052

[27] Okreglak V, Drubin DG. Loss of Aip1 reveals a role in maintaining the actin monomer pool and an in vivo oligomer assembly pathway. J Cell Biol. 2010;188(6):769–77. Epub 2010/03/17. 10.1083/jcb.200909176 20231387 PMC2845081

[28] Lechuga S, Baranwal S, Ivanov AI. Actin-interacting protein 1 controls assembly and permeability of intestinal epithelial apical junctions. American journal of physiology Gastrointestinal and liver physiology. 2015;308(9):G745–56. Epub 2015/03/21. 10.1152/ajpgi.00446.2014 25792565 PMC4421013

[29] Luxenburg C, Heller E, Pasolli HA, Chai S, Nikolova M, Stokes N, et al Wdr1-mediated cell shape dynamics and cortical tension are essential for epidermal planar cell polarity. Nat Cell Biol. 2015;17(5):592–604. Epub 2015/04/29. 10.1038/ncb3146 25915128 PMC4523270

[30] Xue B, Robinson RC. Guardians of the actin monomer. European journal of cell biology. 2013;92(10–11):316–32. Epub 2013/11/26. 10.1016/j.ejcb.2013.10.012 . 24268205

[31] Ciobanasu C, Faivre B, Le Clainche C. Reconstituting actomyosin-dependent mechanosensitive protein complexes in vitro. Nature protocols. 2015;10(1):75–89. Epub 2014/12/17. 10.1038/nprot.2014.200 . 25502885

[32] del Rio A, Perez-Jimenez R, Liu R, Roca-Cusachs P, Fernandez JM, Sheetz MP. Stretching single talin rod molecules activates vinculin binding. Science. 2009;323(5914):638–41. Epub 2009/01/31. 10.1126/science.1162912 . 19179532 PMC9339221

[33] Humphries JD, Wang P, Streuli C, Geiger B, Humphries MJ, Ballestrem C. Vinculin controls focal adhesion formation by direct interactions with talin and actin. J Cell Biol. 2007;179(5):1043–57. Epub 2007/12/07. 10.1083/jcb.200703036 18056416 PMC2099183

[34] Calderwood DA, Zent R, Grant R, Rees DJ, Hynes RO, Ginsberg MH. The Talin head domain binds to integrin beta subunit cytoplasmic tails and regulates integrin activation. J Biol Chem. 1999;274(40):28071–4. Epub 1999/09/25. . 10497155 10.1074/jbc.274.40.28071

[35] Wegener KL, Partridge AW, Han J, Pickford AR, Liddington RC, Ginsberg MH, et al Structural basis of integrin activation by talin. Cell. 2007;128(1):171–82. Epub 2007/01/16. 10.1016/j.cell.2006.10.048 . 17218263

[36] Lefebvre P, White JG, Krumwiede MD, Cohen I. Role of actin in platelet function. European journal of cell biology. 1993;62(2):194–204. Epub 1993/12/01. . 7925478

[37] Bennett JS, Zigmond S, Vilaire G, Cunningham ME, Bednar B. The platelet cytoskeleton regulates the affinity of the integrin alpha(IIb)beta(3) for fibrinogen. J Biol Chem. 1999;274(36):25301–7. Epub 1999/08/28. . 10464255 10.1074/jbc.274.36.25301

[38] Hemmings L, Rees DJ, Ohanian V, Bolton SJ, Gilmore AP, Patel B, et al Talin contains three actin-binding sites each of which is adjacent to a vinculin-binding site. Journal of cell science. 1996;109 (Pt 11):2715–26. Epub 1996/11/01. . 8937989 10.1242/jcs.109.11.2715

[39] Muguruma M, Matsumura S, Fukazawa T. Direct interactions between talin and actin. Biochem Biophys Res Commun. 1990;171(3):1217–23. Epub 1990/09/28. . 2121138 10.1016/0006-291x(90)90815-5

[40] Ciobanasu C, Faivre B, Le Clainche C. Actomyosin-dependent formation of the mechanosensitive talin-vinculin complex reinforces actin anchoring. Nature communications. 2014;5:3095 Epub 2014/01/24. 10.1038/ncomms4095 24452080 PMC3916842

